# Evaluation of Aldosterone Suppression by Cinnarizine, a Putative Cav1.3 Inhibitor

**DOI:** 10.1210/clinem/dgaf081

**Published:** 2025-02-10

**Authors:** Elisabeth Ng, Yun-Ni Lee, Angela Taylor, Fozia Shaheen, Elena Azizan, William M Drake, Morris J Brown

**Affiliations:** Clinical Pharmacology & Precision Medicine, William Harvey Research Institute, Faculty of Medicine and Dentistry, Queen Mary University of London, Charterhouse Square, London EC1M 6BQ, UK; Department of Endocrinology, St Bartholomew's Hospital, Barts Health, London EC1A 7BE, UK; Clinical Pharmacology & Precision Medicine, William Harvey Research Institute, Faculty of Medicine and Dentistry, Queen Mary University of London, Charterhouse Square, London EC1M 6BQ, UK; Department of Endocrinology, St Bartholomew's Hospital, Barts Health, London EC1A 7BE, UK; Steroid Metabolome Analysis Core (SMAC), Institute of Metabolism and Systems Research, University of Birmingham, Birmingham B15 2TT, UK; Steroid Metabolome Analysis Core (SMAC), Institute of Metabolism and Systems Research, University of Birmingham, Birmingham B15 2TT, UK; Department of Medicine, Faculty of Medicine, The National University of Malaysia (UKM) Medical Centre, Selangor 43600, Malaysia; Clinical Pharmacology & Precision Medicine, William Harvey Research Institute, Faculty of Medicine and Dentistry, Queen Mary University of London, Charterhouse Square, London EC1M 6BQ, UK; Department of Endocrinology, St Bartholomew's Hospital, Barts Health, London EC1A 7BE, UK; Clinical Pharmacology & Precision Medicine, William Harvey Research Institute, Faculty of Medicine and Dentistry, Queen Mary University of London, Charterhouse Square, London EC1M 6BQ, UK; Department of Endocrinology, St Bartholomew's Hospital, Barts Health, London EC1A 7BE, UK

**Keywords:** primary aldosteronism, calcium channel blocker, aldosterone, cinnarizine, nifedipine

## Abstract

**Context:**

Primary aldosteronism (PA) is commonly caused by somatic mutations of *CACNA1D* encoding Cav1.3, one of the four L-type calcium channels. The over-the-counter drug, cinnarizine, fits the Cav1.3 crystal structure pore domain.

**Objective:**

We hypothesized that Cav1.3 blockade by cinnarizine may achieve similar, or greater, reduction in aldosterone secretion than nonselective Cav1.2/1.3 blockade by nifedipine.

**Methods:**

Separate wells of angiotensin II–stimulated HAC15 cells were treated with either cinnarizine (1-30 μM) or nifedipine (1-100 μM). Aldosterone concentrations were measured in culture medium; RNA extraction and quantitative polymerase chain reaction were performed to evaluate *CYP11B2* expression. A prospective, open-label, crossover study was conducted of 15 adults with PA, treated with 2 weeks of cinnarizine 30 mg 3 times a day or nifedipine extended release 60 mg daily, separated by a 2-week washout. The hierarchical primary outcome was change in aldosterone-to-renin ratio (ARR), urinary tetrahydroaldosterone (THA), and plasma aldosterone concentration (PAC). Blood pressure change was a secondary outcome. Parametric analysis was undertaken on log-transformed data. (ClinicalTrials.gov: NCT05686993)

**Results:**

Both drugs reduced aldosterone concentrations and *CYP11B2* expression in vitro. Mean changes ± SEM in fold change of aldosterone concentrations and *CYP11B2* were −0.47 ± 0.05 and −0.56 ± 0.07, respectively, with cinnarizine 30 μM and −0.59 ± 0.05 and −0.78 ± 0.07 with nifedipine 100 μM. In the clinical crossover trial, ARR was reduced by nifedipine but not cinnarizine (F = 3.25; *P* = .047); PAC rose with both drugs (F = 4.77; *P* = .013), but urinary THA was unchanged.

**Conclusion:**

A Cav1.3 ligand, cinnarizine, reduced aldosterone secretion from adrenocortical cells, but at maximum-soluble concentrations was less effective than the nonselective calcium blocker, nifedipine. At clinical doses, cinnarizine did not reduce plasma ARR in patients with PA, and, as in vitro, was inferior to nifedipine. The limited efficacy of high-dose nifedipine may be due to incomplete Cav1.3 blockade, or to a role for non–L-type calcium channels in aldosterone secretion.

Hypertension affects more than 1 in 4 adults and 5% to 15% of people with hypertension have primary aldosteronism (PA) (1). People with PA have higher risks of cardiovascular and metabolic disease when compared to their blood pressure (BP)-matched counterparts with essential hypertension (2). It is estimated that less than 2% of patients with PA are detected and therefore treated (3). PA was traditionally divided into unilateral and bilateral; however, there is increasing recognition that PA falls on a spectrum, and it can be multifocal and asymmetrical (4). The management of unilateral PA is surgery to remove the culprit gland, while bilateral PA is treated with long-term drug therapy, typically mineralocorticoid receptor antagonists (MRA).

Aldosterone-producing adenomas (APA) causing PA have been identified to have specific genetic mutations, including more than 70 reported in *CACNA1D*, which encodes the calcium channel Cav1.3 (5-8). Some somatic mutations in APAs correlate with distinct phenotypes (7, 9-11). These phenotypes can include different outcomes after adrenalectomy. In several studies *CACNA1D* mutations appear to be associated with a lower chance of biochemical and clinical cure, and a greater likelihood of persistent disease requiring medical therapy (12, 13). On the other hand, the *KCNJ5* mutations (encoding for a potassium channel) were associated with a higher chance of cure after surgery (12, 14). Additionally, in a multi-institution cohort of adrenal glands with idiopathic hyperplasia, most (14/15) had the *CACNA1D* mutation (15). This contributes to the hypothesis that the *CACNA1D* mutations are present in asymmetric rather than truly unilateral PA (16), and that effective medical therapy may be preferable to adrenalectomy if surrogate predictors of *CACNA1D* mutations can be ascertained. The *CACNA1D* mutation has been more commonly observed in male patients with APA, and is the most frequently mutated aldosterone-driver gene in people of Black ethnicity (17).

In addition to existing and emerging therapies yet to be evaluated in PA (18, 19), the discovery of *CACNA1D* mutations in APA suggested potential therapeutic value of selective Cav1.3 inhibition. Dihydropyridine and other calcium channel blockers used for the treatment of hypertension are nonselective between Cav1.3 and the 75% homologous, vascular Cav1.2 (20). Patients with PA are commonly treated with calcium channel blockade to lower BP, but the effect on plasma aldosterone concentrations (PAC) as a contribution to BP reduction has not been well described. Selective Cav1.3 blockade should avoid the dose-limiting side effects of nonselective L-type calcium channel blockers, such as peripheral edema (21, 22), allowing greater aldosterone suppression at maximum dose (23). Previous work identified compound 8 as a highly selective Cav1.3 inhibitor, with the ability to suppress aldosterone and cause programmed cell death of adrenocortical cells; however, some of its effects may have been limited to the subtypes of β and δ units expressed in the stable cells used for testing (23, 24). A nonvascular calcium channel blocker, cinnarizine, fits the crystal structure pore domain of Cav1.3 (25). We speculated that its clinical use, to reduce vestibular symptoms, without documented effects on BP, might reflect efficacy at well-studied Cav1.3 receptors in the vestibular inner hair cells (26) without actions at vascular Cav1.2 receptors.

The aims of our studies were to test whether, 1) in adrenocortical cells expressing more Cav1.3 than Cav1.2, cinnarizine (proposed Cav1.3 inhibitor) was more effective than nifedipine (nonselective Cav1.2/Cav1.3 inhibitor) in inhibiting aldosterone production, and 2) in a proof-of-concept clinical study in people with PA, cinnarizine may be superior to nifedipine in reducing aldosterone production, estimated as aldosterone-to-renin ratio (ARR) and 24-hour urinary tetrahydroaldosterone (THA) excretion.

## Materials and Methods

Evaluation of cinnarizine and nifedipine in vitro and ex vivo was performed on HAC15 cells, a human adrenocortical cell line, and on primary human cell cultures from adrenal tissues of patients operated on for PA. An in vivo pilot study was performed comparing cinnarizine to nifedipine in adults with PA.

### Primary Cell Culture

Adrenal tissue was collected directly from surgery of patients undergoing adrenalectomy for PA, with samples identified as part of the tumor (APA) or adjacent adrenal tissue and handled separately for the entire subsequent process. Available tissue was collected in culture medium and transported to the laboratory on ice for dissociation within 1 to 2 hours of retrieval. After dissection to remove surrounding fat and vessels, the tissue was digested in collagenase from *Clostridium histolyticum* (Sigma-Aldrich, C9697) for 3 to 4 hours, washed twice with phosphate-buffered saline, and resuspended in culture medium.

One experiment was performed on a sample collected from a surgery performed on a 56-year-old woman of Black ethnicity with cosecretion of aldosterone and cortisol and a 29 mm adrenal adenoma; her mutation is unknown. A separate crossover experiment was performed on a sample of adrenal tissue adjacent to an APA that was later genotyped and confirmed to have the *CACNA1D* mutation. Cells were treated first with either cinnarizine 10 μM or nifedipine 10 μM and after 24 hours of treatment, medium was harvested and a subset of wells processed for RNA extraction (n = 2). Remaining wells (n = 4) underwent a phosphate-buffered saline wash and a preincubation period (48 hours) in complete medium before being serum-starved in nonsupplemented medium for 4 hours before treatment with the alternative drug.

### Cell Culture Experiments

Cell culture experiments were performed on HAC15 cells generously provided by Prof Celso Gomez-Sanchez (27). HAC15 cells originated as a subclone of H295R cells, and our stock has the p.Ser45Pro mutation of *CTNNB1* reported in the parent cell line (27). Both HAC15 and primary adrenal cells were maintained in Dulbecco's Modified Eagle Medium (DMEM)/Nutrient Mixture F-12 medium supplemented with 10% fetal bovine serum, 1% insulin-transferrin-selenium (100×) and 1% L-Glutamine-Penicillin-Streptomycin solution (L-glutamine 200 mM, streptomycin 10 mg/mL, penicillin 10 000 units). HAC15 cells were seeded at 3.0 × 10^5^ cells per well for 24 hours before treatment. Primary adrenal cells were seeded 5 to 7 days after dissociation at a density between 1.5 and 2.0 × 10^5^ cells per well depending on availability of tissue. All treatments were performed using serum-free nonsupplemented medium. Cells were serum-starved in nonsupplemented medium for 4 hours before treatment. They were then maintained in treatment conditions for 24 hours before collection of medium for analysis of aldosterone concentrations and collection of the cell pellet for RNA extraction.

Cinnarizine and nifedipine (Sigma-Aldrich) were reconstituted in dimethyl sulfoxide (DMSO) to stock concentrations of 8 and 100 mM, respectively (stock concentration of cinnarizine limited by solubility). Stock concentrations were further diluted in sterile, unsupplemented DMEM/Nutrient F-12 Ham for treatments, with the same proportion of DMSO used in all treatment groups (4/1000). Cinnarizine was used at doses of 1, 3, 10, and 30 μM, and nifedipine at 1, 10, and 100 μM. Treatments in HAC15 cells were performed in the presence of Angiotensin II (Ang II) stimulation at a dose of 10^−8^ M. Each experiment included an untreated group with diluent only to confirm cell responsiveness to Ang II. Experiments were conducted with technical and biological triplicates for quantitative polymerase chain reaction (qPCR) and aldosterone concentration measurement.

### RNA Extraction and Quantitative Polymerase Chain Reaction

RNA extraction was performed using the PureLink RNA Mini Kit (Invitrogen, 12183018A) according to the manufacturer’s instructions. RNA was reverse-transcribed to complementary DNA using a High-Capacity RNA-to-cDNA Kit (Applied Biosystems, 4387406). qPCR was performed to assess for *CYP11B2* messenger RNA (mRNA) expression, using *18S* as the housekeeping gene. TaqMan assay probes were used to quantify the gene of interest and the ΔΔ CT analysis method was used to quantify gene expression (ThermoFisher, assay ID Hs01597732_m1 for *CYP11B2* and Hs99999901_s1 for *18S*).

### Aldosterone Assay

Aldosterone concentrations were measured in culture medium using an aldosterone assay kit (Cisbio, 64ALDPEH). Fluorescent signals were read at 665 and 625 nM with the FLUOstar Omega plate reader (BMG Labtech).

### RNA Sequencing Expression of Genes Encoding Calcium Channels

Extracted RNA from H295R cells (from which HAC15 cells are a subclone) was sequenced by the Barts and the London Genome Center, Blizard Institute in London, with quality control performed using an Agilent RNA 6000 nano reagent kit and Agilent 2100 bioanalyzer (12). Sequencing was performed on Illumina's NextSeq 500 system, using the high-output 150-cycle kit v2.5 and bcl2fastq Conversion Software. These data were retrieved from the nontransfected cells of an extensive study performed on the effects of the *CADM1* mutations; methodology of processing and analysis has been previously described (28). In addition, RNA sequencing (RNASeq) data of functional adenomas and adjacent adrenal tissue from 64 patients with PA who underwent adrenalectomies as part of the MATCH study were evaluated (12). RNASeq data from the H295R cells and human adrenal samples were evaluated to calculate the mean and SD for genes encoding calcium channels in the adrenal cell line, tumor tissue, and adrenal tissue adjacent to tumor. This includes Cav1.3 (encoded by *CACNA1D*), Cav1.2 (encoded by *CACNA1C*), and Cav3.2 (encoded by *CACNA1H*).

### Pilot Study of Cinnarizine Compared to Nifedipine in Adults With Primary Aldosteronism

#### Study design

This was a pilot, prospective, open-label study of adults with PA, designed to compare the change in ARR, plasma aldosterone, and urinary THA concentrations with cinnarizine and nifedipine treatment. Patients were recruited from a single site in London, UK (St Bartholomew's Hospital). The study was conducted in accordance with the principles of the Declaration of Helsinki. The study protocol was approved by the National Research Ethics Service (Hampshire A Research Ethics Committee; Integrated Research Application System project ID 319634) and registered at ClinicalTrials.gov (NCT05686993).

#### Participants

Participants were recruited through the endocrinology clinic at St Bartholomew's Hospital. The criteria for inclusion were age 18 years or older who were able and willing to give informed consent, with confirmed PA according to the Endocrine Society guidelines (29). Patients with clinical features suggestive of an increased likelihood of a *CACNA1D* mutation were favored; these features included Black ethnicity, male sex (17), and previous adrenalectomy without cure from PA. Exclusion criteria were uncontrolled hypertension requiring use of MRA; inability or unwillingness to provide consent; allergy to cinnarizine or nifedipine or their excipients; existing use of cinnarizine or nifedipine for an alternative indication; breastfeeding or pregnant women; diagnosis of Parkinson disease; severe hepatic or renal insufficiency; concurrent use of sedating central nervous system depressants or rifampicin; porphyria; cardiogenic shock; clinically significant aortic stenosis; unstable angina; a history of myocardial infarction within the previous month; or previous gastrointestinal or esophageal obstruction or ileostomy. All participants provided written informed consent prior to study participation.

#### Drug treatments

Participants were randomly assigned (30) to start with either cinnarizine 30 mg 3 times a day or nifedipine LA (Adalat LA) 60 mg daily for 2 weeks each, with a 2-week washout period in between. On initial enrollment, participant medications were assessed and medications that interfere with the renin-angiotensin-aldosterone system or were calcium channel blockers were discontinued and replaced with moxonidine, doxazosin, and/or hydralazine. These agents were titrated during the washout period to achieve a BP less than 140/90 mm Hg, with a view to keep all non-study BP-lowering agents at the same dose and frequency from commencement of the first study drug, and for at least 6 weeks thereafter, unless adverse effects developed or progressed from a tolerable state. Spironolactone, eplerenone, and all other interfering agents were discontinued for a minimum of 6, 4, and 2 weeks, respectively, prior to commencement of the first study drug.

#### Blood pressure

BP assessment was undertaken at each clinic visit. Participants were rested for 5 to 10 minutes and 3 BP readings were then taken from the right arm. Participants also documented home BP readings daily, with advice given to measure BP 3 times after 5 to 10 minutes of rest.

#### Adverse events

Adverse events were assessed at each study review with history-taking and examination. Participants were given a mobile contact number and email address to report any adverse events during the course of the study. The plan for serious adverse events was reporting within 24 hours to the principal investigator, sponsor and research ethics committee.

#### Blood and urine tests

Participants underwent one 24-hour urine collection and 2 sets of blood tests (on separate days at the same approximate time) before and after each study drug. Pretreatment blood and urine tests were collected at the end of the washout period while the participant remained on noninterfering medications. Posttreatment blood and urine tests were collected while the participant was still taking the study treatment, after at least 13 days (eg, on day 13 and 14 of the study medication or later). Urine studies were collected over a 24-hour period, with no specific advice given about salt intake aside from avoiding salt restriction.

Each participant had all their blood and urine tests performed at the same pathology laboratory to allow intraindividual comparison. Most participants had their samples collected at St Bartholomew's Hospital except for 2 participants, who attended their local hospital for study investigations due to logistical issues. PAC was measured by automated chemiluminescence immunoassay (LIAISON; DiaSorin) for all samples analyzed at St Bartholomew's Hospital and one externally processed sample, with the one other external sample analyzed by liquid chromatography–tandem mass spectrometry (LC-MS/MS). Plasma renin activity was analyzed by LC-MS/MS (North West London Pathology) for all but one sample, where direct renin concentration was measured by automated chemiluminescence immunoassay (LIAISON; DiaSorin). This renin result measured as direct renin concentration was converted to plasma renin activity to allow comparison, using a commonly applied conversion factor of 8.2 (29).

Urine samples collected from participants were sent for local processing of urine electrolytes and volume. A 15-mL aliquot was taken beforehand and stored at −80 °C for analysis of urinary THA. These samples were batch-transferred on dry ice to the University of Birmingham for analysis by LC-MS/MS as previously described (12, 31).

#### Statistical analysis

qPCR and aldosterone assay data are presented as mean ± SEM; data were compared using a one-way analysis of variance (ANOVA) and Bonferroni multiple comparisons test where more than 2 groups were present, and an unpaired *t* test when comparing 2 groups only. RNASeq reads were presented as mean (SD), and differences in reads between calcium channels were compared using paired *t* tests. Statistical analysis was performed using GraphPad Prism. A *P* value less than .05 was considered statistically significant.

The hierarchical primary outcome was change in ARR, urinary aldosterone, and PAC before and after both study medications. The secondary outcome was change in BP with both study medications. A sample size of 15 participants was calculated before study commencement. A 30% reduction in plasma aldosterone was postulated, which was the reduction on the lowest dose of a first-in-class direct aldosterone synthase inhibitor (ASI) (18). The SD of change in aldosterone in that study was 40% of the baseline value. At α .05 and β .09, a one-sample size of 19 was calculated. By collecting 2 baseline values for aldosterone, and measuring aldosterone twice on each treatment, a repeated-measures ANOVA was planned, with an estimated reduction of sample size of 44% for each duplicate (32).

## Results

### Evaluation of Cinnarizine and Nifedipine In Vitro and Ex Vivo

#### In Vitro

**Figure 1.**
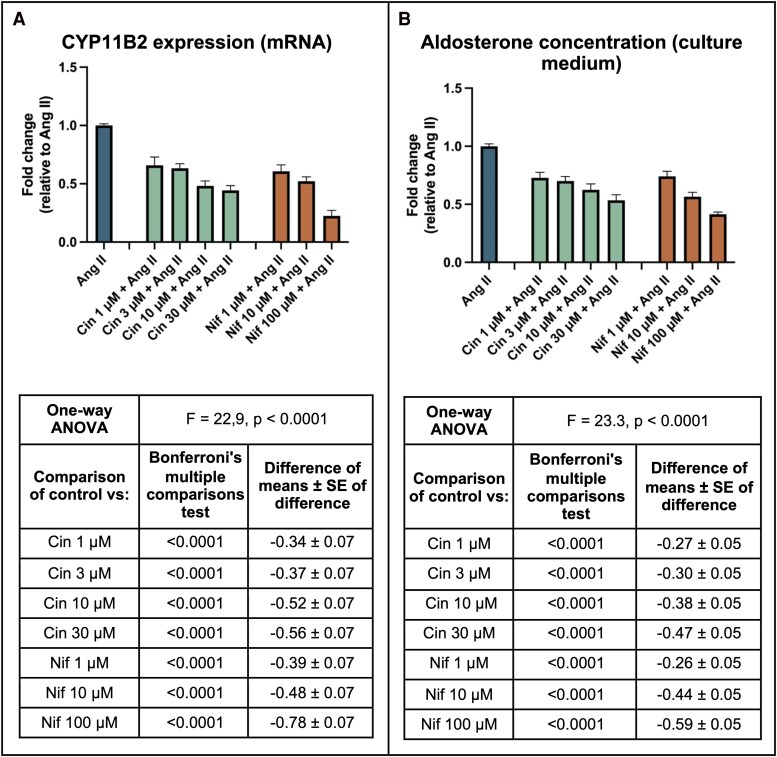
*CYP11B2* expression and aldosterone concentration (fold change) in HAC15 cells in response to treatment with cinnarizine (Cin) and nifedipine (Nif). A, *CYP11B2* expression (messenger RNA [mRNA]) fold change and B, aldosterone concentration fold change, in response to treatment with Cin and Nif compared to control group (Ang II only). All treatment groups contained Ang II at a dose of 10^−8^ M, and all groups contained DMSO as the drug diluent at a concentration of 4:1000. Abbreviations: Ang II, angiotensin II; ANOVA, analysis of variance; Cin, cinnarizine; Nif, nifedipine; qPCR, quantitative polymerase chain reaction.

Treatment of HAC15 cells with cinnarizine demonstrated a dose-related reduction in CYP11B2 expression (Fig. 1A and Supplementary Fig. S1 (33)). A dose of cinnarizine 30 μM caused a fold-change reduction of 0.56 ± 0.07 (*P* < .0001) in CYP11B2 expression and 0.47 ± 0.05 in aldosterone concentrations (*P* < .0001) (see Fig. 1A). A dose-related reduction in CYP11B2 expression was also seen in response to nifedipine (Fig. 1A), with a fold-change reduction of 0.78 ± 0.07 with 100 μM and 0.48 ± 0.07 with 10 μM (*P* < .001 for both compared to Ang II alone). A similar trend was seen with the fold change in aldosterone concentrations (Fig. 1B). Ang II responsiveness was confirmed by a doubling of CYP11B2 RNA expression and aldosterone concentration, comparing Ang II (10^−8^ M) stimulation with vehicle (equivalent DMSO only).

#### Ex vivo

**Figure 2.**
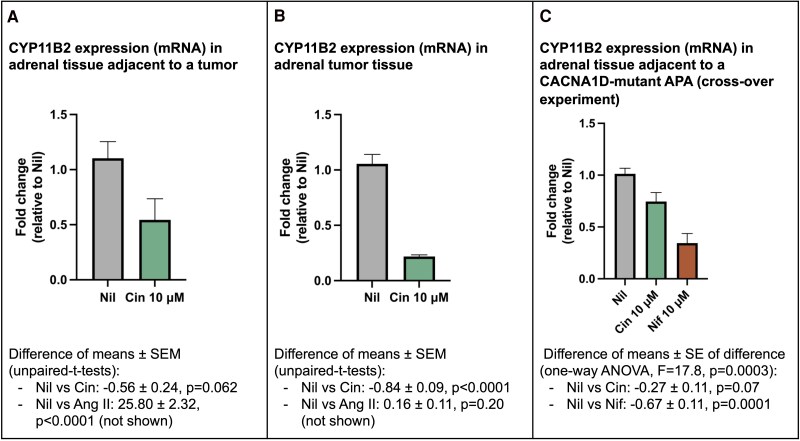
*CYP11B2* expression in primary adrenal cell culture in response to treatment with cinnarizine (Cin) and nifedipine (Nif). *CYP11B2* expression (messenger RNA [mRNA]) as fold change in A, tissue adjacent to an aldosterone-producing adenoma (APA) and B, in the APA (tumor) itself. C, Crossover experiment using Cin followed by Nif (and vice versa) in adrenal tissue adjacent to a *CACNA1D* mutant APA. Abbreviations: Ang II, angiotensin II; APA, aldosterone-producing adenoma; Cin, cinnarizine; Nif, nifedipine.

In primary human adrenal cells cultured from an APA and adjacent adrenal of a patient with unilateral PA (mutation unknown), cinnarizine 10^−5^ M caused a 0.84 ± 0.09 (*P* < .0001) fold-change reduction of *CYP11B2* expression in tumor, while in adjacent tissue the fold-change reduction was 0.56 ± 0.24 (*P* = .06) (Fig. 2).

Results of the crossover experiment performed in tissue adjacent to a *CACNA1D*-mutant tumor are shown in Fig. 2C. There was a reduction in *CYP11B2* expression with nifedipine (fold-change reduction 0.67 ± 0.11; *P* = .0001) but not cinnarizine (fold-change reduction 0.27 ± 0.11; *P* = .07).

#### RNA Sequencing

In H295R cells, gene expression as normalized reads on RNASeq (mean (SD)) were 6.0 (0.2) for *CACNA1C* and 16.3 (0.5) for *CACNA1D* (*P* < .0001). In APA, the reads for *CACNA1C* were 16.2 (14.3) and for *CACNA1D* 93.2 (41.3) (*P* < .0001). In adjacent tissue, this was 5.9 (3.9) for *CACNA1C* and 48.0 (17.4) for *CACNA1D* (*P* < .0001). There was also substantial expression of *CACNA1H,* encoding the T-channel Cav3.2, which in human adrenal samples exceeded the sum of all other calcium channels (140.6 [87.5] in tumor and 66.5 [34.1] in adjacent adrenal tissue). Reads for differential expression of all genes encoding calcium channels are shown in Supplementary Table S1 (33).

### Pilot Study of Cinnarizine Compared to Nifedipine in Adults With Primary Aldosteronism

#### Participant characteristics

**Figure 3.**
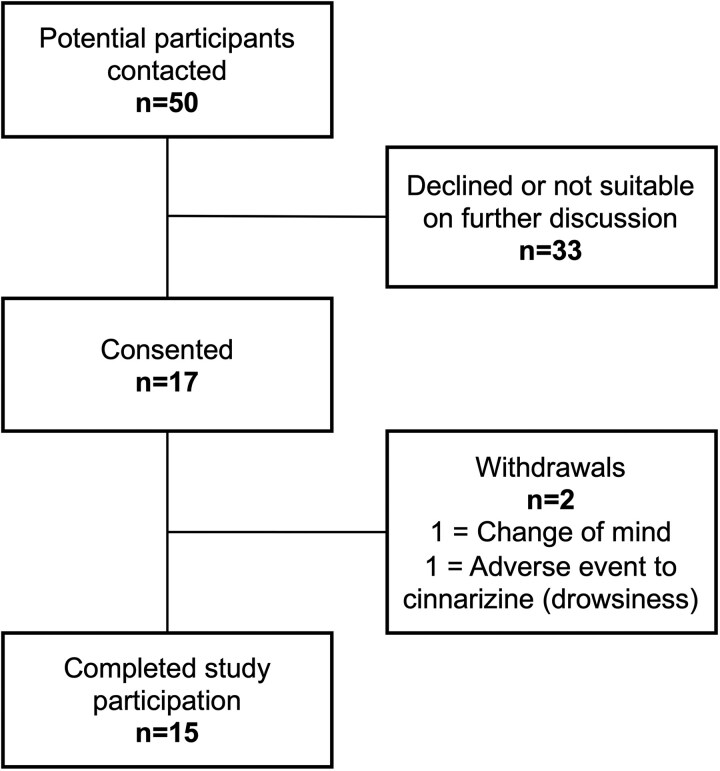
Flow diagram of recruitment and enrollment.

There were 17 participants recruited between May 5 and September 1, 2023. Two participants withdrew, one for personal reasons and the other after experiencing drowsiness on day 2 of treatment with cinnarizine. A total of 15 patients completed the study, fulfilling the proposed sample size (Fig. 3). Participant characteristics are shown in Table 1. Most were male (13/15), mean age was 55 ± 13 years, mean body mass index was 31.9 ± 3.7, and 10 of 15 were of Black ethnicity. Mean duration of hypertension was 12 years and 6 of 15 had a previous adrenalectomy with subsequent persistent or recurrent aldosterone excess.

**Table 1.** Participant characteristics

Characteristic	N = 15
Sex	
Female	2/15 (13%)
Male	13/15 (87%)
Age, years	54.7 (12.8)
Weight, kg	99.5 (12)
Height, cm	176.8 (8.2)
Body mass index, kg/m^2^	31.9 (3.7)
Ethnicity	
Black or African American	10/15 (67%)
White	5/15 (33%)
Age at onset of hypertension, years	38.1 (8)
Duration of hypertension, years	12 (8, 29)
Number of comorbidities	2.4 (1.8)
Number of antihypertensive medications at baseline	1 (1-3)
Previous adrenalectomy	6/15 (40%)
Adrenal vein sampling subtype (bilateral / unilateral)	9 (60%)/5 (33%)*

Data presented as n/N (%) for categorical data; median (Q1-Q3) for nonparametric continuous data; mean (SD) for parametric continuous data. *One patient not cannulated bilaterally.

#### Change in biochemistry with cinnarizine and nifedipine

**Figure 4.**
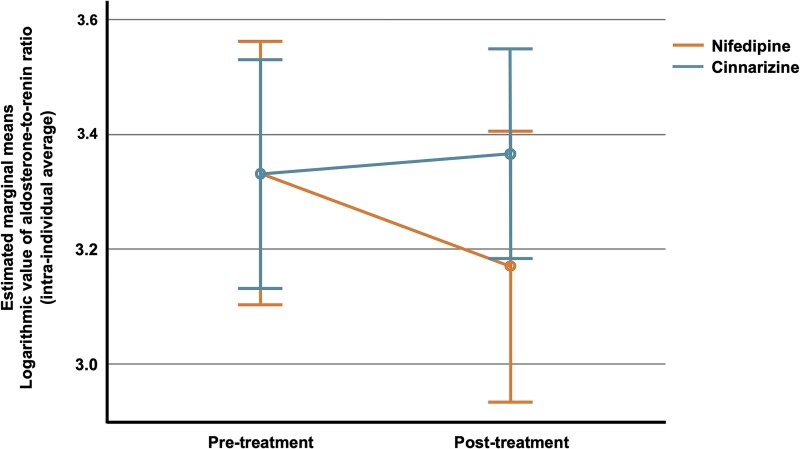
Repeated measures analysis of variance of the log value of average aldosterone-to-renin ratio pre treatment compared to post treatment.

Nifedipine reduced ARR from 2870 (1029-4205) to 1808 (687-3130) pmol/L:nmol/L/h (median (Q1 - Q3)) but ARR increased with cinnarizine, from 2458 (922-3776) to 2739 (1667-3816) pmol/L:nmol/L/h (*F* = 3.25; *P* = .0468; repeated-measures ANOVA) (Table 2 and Fig. 4). PAC increased both with cinnarizine and nifedipine (*F* = 4.77; *P* = .013), while renin increased with nifedipine and decreased with cinnarizine (*F* = 11.77; *P* = .0001) (Table 2). Electrolytes and renal function remained stable with both study medications, with no change in sodium, potassium, creatinine, or estimated glomerular filtration rate.

**Table 2.** Aldosterone, renin, aldosterone-to-renin ratio, and urinary tetrahydroaldosterone before and after each study drug

	Aldosterone, pmol/L	Renin, ng/mL/h	ARR, pmol/L:ng/mL/h	Urinary THA, mcg/24 h
**Pre nifedipine**	636 (383-1044)	0.25 (0.19-0.40)	2870 (1029-4205)	109 (58-168)
**Post nifedipine**	765 (442-1255)	0.50 (0.35-0.80)	1808 (687-3130)	84 (44-148)
**Pre cinnarizine**	505 (380-809)	0.25 (0.19-0.40)	2458 (922-3776)	111 (65-186)
**Post cinnarizine**	649 (482-877)	0.20 (0.20-0.40)	2739 (1667-3816)	98 (74-166)
*P* value*^a^*	.0113	.0001	.0468	.5014

Data presented as median (Q1-Q3) for nonparametric continuous data.

Abbreviations: ANOVA, analysis of variance; ARR, aldosterone-to-renin ratio; THA, tetrahydroaldosterone.

^a^Repeated-measures ANOVA performed on log values across 4 time points with comparison of means between pre and post study drug (log values passed normality test).

#### Change in urinary profile with cinnarizine and nifedipine

Urinary THA decreased slightly with nifedipine and increased with cinnarizine (see Table 2), with no difference when compared using a repeated measures ANOVA test on log values (*F* = 0.75; *P* = .50). Urinary volume and potassium did not change with either treatment, but urinary sodium did decrease with nifedipine (mean 101.3 to 89.9 mmol/day) and increase with cinnarizine (73.0 to 84.9 mmol/day) (*F* = 3.21; *P* = .046).

#### Change in blood pressure with cinnarizine and nifedipine

Systolic blood pressure fell by 18 ± 13 mm Hg with nifedipine (*P* = .0001) and 6 ± 14 mm Hg with cinnarizine (*P* = .112). Diastolic blood pressure fell by 8 ± 10 mm Hg with nifedipine (*P* = .009) and by 5 ± 10 mm Hg with cinnarizine (*P* = .065).

Adverse effects both to cinnarizine and nifedipine are shown in Table 3. The most common adverse effect with cinnarizine was drowsiness, with this reported to be present in the initial 48 to 72 hours of treatment before resolution. There were more adverse effects reported with nifedipine, with headache being the most common.

**Table 3.** Adverse effects experienced by the 15 study participants

Adverse effect	Nifedipine	Cinnarizine
Abdominal pain	0	0
Drowsiness	0	4 (29.7%)
Nausea	1 (6.7%)	0
Vomiting	0	0
Dry mouth	1 (6.7%)	0
Fatigue	3 (20%)	2 (13.3%)
Headache	4 (29.7%)	2 (13.3%)
Dizziness	3 (20%)	2 (13.3%)
Flushing	0	0
Palpitations	2 (13.3%)	2 (13.3%)
Peripheral edema	2 (13.3%)	0
Constipation	1 (6.7%)	0
Other	0	0

## Discussion

Current medical options for the large proportion of PA patients not suitable for adrenalectomy are limited. Spironolactone has well-documented side effects, and it is not certain whether mineralocorticoid blockade fully prevents the damaging consequences of the combined aldosterone and sodium excess that characterizes PA. The MR is expressed in multiple tissues, whose blockade may not always lead to desirable consequences. Just as it has been useful to have a choice that blocks the synthesis and actions of angiotensin (respectively angiotensin-converting enzyme inhibitors and angiotensin receptor blockers), so it may prove useful to target aldosterone with drugs that suppress its production. ASI promise an alternative strategy for the treatment of PA but at present most of the published evidence is in “resistant” hypertension. It was against this background that we explored the possibility of “repurposing” an established drug with a favorable toxicology profile (cinnarizine) for use as a possible selective Cav1.3 inhibitor, hypothesizing that it would lower aldosterone production in vitro and in vivo. This study has shown that both cinnarizine and nifedipine lowered *CYP11B2* and aldosterone levels in vitro and ex vivo, while in vivo nifedipine lowered ARR and urinary THA but cinnarizine did not. The clinical trial did not show a statistically significant reduction in aldosterone concentration with either drug. BP reduction was seen with nifedipine but not cinnarizine. Cinnarizine was generally well tolerated while the expected adverse effects with nifedipine were seen.

Nifedipine was used as the comparator to cinnarizine given its modest Cav1.2 selectivity (34), our previous demonstration that it inhibits ex vivo secretion of aldosterone (23), and its established safety and efficacy in hypertension (22). As expected from its vascular, Cav1.2, inhibition, nifedipine increases cardiopulmonary baroreceptor sympathetic sensitivity (35), which increases renal afferent arteriolar constriction and therefore renin secretion, in turn increasing aldosterone secretion (36). Unlike its inhibition of aldosterone production in vitro (37), multiple studies in essential hypertension have been inconsistent in their findings on the effect of nifedipine on aldosterone levels, reporting either an increase (38-40), reduction (41, 42), or no change (43). MRA are the mainstay of medical management in PA and some, such as finerenone (44), have been derived from dihydropyridine-based compounds. Normalization of renin is the goal with MR antagonism in PA (45) but at the cost of an increase in plasma aldosterone. This may not matter if all its receptors are blocked, but there is continued uncertainty whether aldosterone may stimulate a nongenomic receptor, putatively encoded by *GPER* (46). In our study nifedipine increased renin and lowered ARR. The potential for a disease-specific benefit to this agent beyond an antihypertensive effect clearly requires validation in a larger study and evaluation of the effect on long-term cardiovascular outcomes.

The RNASeq data show higher expression of *CACNA1D* (Cav1.3) than *CACNA1C* (Cav1.2) in the H295R cell line, human APA, and adjacent adrenal tissue (see Supplementary Table S1 (33)). On the other hand, inspection of our single-nucleus data from 3 *CACNA1D*-mutant APAs shows the upregulated expression of *CACNA1C* to be primarily in *CYP11B2*-expressing, not vascular cells (47), and this upregulation may contribute to a lower potential of selective Cav1.3 blockade to inhibit aldosterone secretion in patients with PA than from an adrenocortical cell line. Another finding from the RNASeq data was high expression of Cav 3.2, encoded by *CACNA1H*, a low-voltage T channel. These channels would not be blocked by nifedipine except at very high concentrations (IC_50_ = 98 μM in transfected HEK293 cells reported by Shcheglovitov et al (48)). This may in part explain the shallow dose-response curves seen with cinnarizine and nifedipine. A previously licensed drug, mibefradil, which blocked T and L channels (49), was more effective than an L-blocker in inhibiting Ang II-, but not K^+^ -, stimulated aldosterone secretion from H295R cells. However, clinical doses of mibefradil did not reduce plasma aldosterone in patients with hypertension (50), and the drug was withdrawn for cardiac toxicity shortly after launch. No subsequent T-channel blocker has been licensed in the United States or Europe (51).

Cinnarizine was used at a dose that would be adequate to reduce vertigo by blocking the vestibular Cav1.3 receptor (52, 53), with a 2-week treatment period selected to minimize the risk of adverse effects. In addition to calcium channel-blocking action, cinnarizine has antihistaminic, antiserotonergic, and antidopaminergic properties (54). The latter leads to a risk of extrapyramidal side effects (55), though this was very rare in postmarketing surveillance studies for cinnarizine (56). No patients in our study experienced extrapyramidal side effects at the dose and duration used, but the antidopaminergic property of cinnarizine may have contributed to the rise in ARR seen with cinnarizine. Dopamine D_2_ receptors inhibit aldosterone secretion, and their blockade by metoclopramide increases plasma aldosterone levels (57).

The limitations of this clinical study are the small sample size, absence of placebo control, and possibly a lack of plasma drug concentrations as an indicator of dose adequacy and absorption. Cinnarizine has low solubility from intestinal media (58), and its gastric solubility varies depending on individual gastric fluid acidity and the fed or fasted state (59). In contrast to cinnarizine, nifedipine is almost completely absorbed from the gastrointestinal tract (60). Differences in absorption and oral bioavailability may have contributed to the different effects seen in vitro vs in vivo. These limitations should be seen in the context of a dearth of prospective studies in PA due in part to the difficulties of maintaining patients off confounding medications, and are offset by our parallel study of the same two drugs, at approximately the same concentrations and doses relative to their IC_50_, both in cells and patients (IC_50_ 1.5 μM for cinnarizine (26) and 2.3 μM for nifedipine (61)). The greater efficacy of nifedipine than cinnarizine, in both our clinical and laboratory studies, but only modest reduction in aldosterone secretion except at concentrations an order of magnitude higher than can be achieved in patients, permit some confidence in the below conclusions. The demographic profile of the participants in the prospective study shows the majority of participants were men and of Black ethnicity; these are traits identified to be more likely seen in people with the *CACNA1D* mutation. Even so, the mutation status of most participants is unknown. This is likely to be the situation in most patients until or unless they come to surgery.

The extent of ex vivo studies was limited by the availability of adrenal tissue, with tissue collected during the course of the study not being found to have the *CACNA1D* mutation. Although it was the discovery of *CACNA1D* mutations that first suggested that a Cav1.3 inhibitor may be beneficial in this subgroup of APA, the clinically relevant hypothesis is likely to be broader given the multiple different sites and electrophysiology of *CACNA1D* mutations, making it unlikely that one drug could target all. The likelihood is that calcium entry is increased in APAs whose mutations depolarize the cell membrane, such that an inhibitor of the cell's main site of calcium entry that may have therapeutic benefit may not be limited to *CACNA1D*-mutant APAs. APA tissue availability was also a limiting factor in the ex vivo crossover experiment given the volume of tissue required; it was therefore performed on adjacent adrenal tissue instead.

Whether limitations of solubility and dosing simply render cinnarizine a less effective calcium blocker than nifedipine at either Cav1.2 or Cav1.3, or whether its limitation is a degree of selectivity for Cav1.3, cinnarizine itself does not appear a viable candidate for repurposing as a drug for PA. On the other hand, we have demonstrated both in vitro and in vivo the ability of nifedipine to inhibit aldosterone production. The degree of inhibition is much less than is seen with the new class of ASI (18, 19). Because of the growing evidence for ethnic variation in the prevalence of *CACNA1D* mutations and the fact that this group of patients may have latent bilateral PA that is not cured by adrenalectomy (12, 17), the development of an effective, selective Cav1.3 inhibitor remains attractive. Historically, the development of ion-channel inhibitors with high selectivity between similar molecules has been challenging. The presence of some Cav1.2 in APAs may place a limit on how much Cav1.3:Cav1.2 is optimal for achieving profound suppression of aldosterone.

In conclusion, although cinnarizine is the only molecule demonstrated to date to sit within the Cav1.3 binding site, maximum concentrations and doses achieved only modest (ex vivo) or no (in vivo) reduction in aldosterone secretion from the adrenals of patients with PA, or in patients with PA, respectively. Nifedipine, which is considered nonselective for Cav1.2 and Cav1.3, was more effective than cinnarizine, but still did not achieve complete (ex vivo) or clinically significant reductions in aldosterone secretion. These results do not exclude the possibility that a more potent, more soluble Cav1.3 inhibitor would have greater efficacy and application than either cinnarizine or nifedipine. However, our findings also caution that other calcium channels not inhibited by therapeutic concentrations of nifedipine may contribute to the stimulation of aldosterone production in PA. A more positive inference from the modest effects of nifedipine on aldosterone and renin is that this class of drug is unlikely to interfere with diagnostic hormone measurements in PA, and that it may be generally easier and safer to interpret results in the presence of a dihydropyridine calcium channel blocker, rather than replacing it with a less effective antihypertensive agent.

## Data Availability

Some or all data sets generated during and/or analyzed during the present study are not publicly available but are available from the corresponding author on reasonable request.

## Clinical Trial Information

ClinicalTrials.gov registration number NCT05686993 (registered January 17, 2023).

## Abbreviations

Ang II: angiotensin II
ANOVA: analysis of variance
APA: aldosterone-producing adenoma
ARR: aldosterone-to-renin ratio
ASI: aldosterone synthase inhibitor
BP: blood pressure
DMSO: dimethyl sulfoxide
LC-MS/MS: liquid chromatography–tandem mass spectrometry
MRA: mineralocorticoid receptor antagonist
mRNA: messenger RNA
PA: primary aldosteronism
PAC: plasma aldosterone concentration
qPCR: quantitative polymerase chain reaction
RNASeq: RNA sequencing
THA: tetrahydroaldosterone
